# The Impact of Macrophage Nucleotide Pools on HIV-1 Reverse Transcription, Viral Replication, and the Development of Novel Antiviral Agents

**DOI:** 10.1155/2012/625983

**Published:** 2012-07-01

**Authors:** Christina Gavegnano, Edward M. Kennedy, Baek Kim, Raymond F. Schinazi

**Affiliations:** ^1^Center for AIDS Research, Laboratory of Biochemical Pharmacology, Department of Pediatrics, Emory University School of Medicine, Atlanta, GA 30022, USA; ^2^Veterans Affairs Medical Center, Atlanta, GA 30033, USA; ^3^Department of Microbiology and Immunology, University of Rochester Medical Center, Rochester, NY 14642, USA

## Abstract

Macrophages are ubiquitous and represent a significant viral reservoir for HIV-1. Macrophages are nondividing, terminally differentiated cells, which have a unique cellular microenvironment relative to actively dividing T lymphocytes, all of which can impact HIV-1 infection/replication, design of inhibitors targeting viral replication in these cells, emergence of mutations within the HIV-1 genome, and disease progression. Scarce dNTPs drive rNTP incorporation into the proviral DNA in macrophages but not lymphocytes. Furthermore, the efficacy of a ribose-based inhibitor that potently inhibits HIV-1 replication in macrophages, has prompted a reconsideration of the previously accepted dogma that 2′-deoxy-based inhibitors demonstrate effective inhibition of HIV-1 replication. Additionally, higher levels of dUTP and rNTP incorporation in macrophages, and lack of repair mechanisms relative to lymphocytes, provide a further mechanistic understanding required to develop targeted inhibition of viral replication in macrophages. Together, the concentrations of dNTPs and rNTPs within macrophages comprise a distinctive cellular environment that directly impacts HIV-1 replication in macrophages and provides unique insight into novel therapeutic mechanisms that could be exploited to eliminate virus from these cells.

## 1. Introduction

Macrophages are a key reservoir for HIV-1, and their ubiquitous nature, multiple, and often independent microenvironments in which they are contained, coupled with their susceptibility to HIV-1 infection [1–3], dictate that further understanding must be garnered about the distinctive characteristics of macrophages and the subsequent impact on the dynamics of HIV-1 infection in these cells. Despite these factors, most of the attention on reservoirs for latent HIV-1 has focused on cells of lymphoid origin, most notably CD4^+^/CD45RO^+^ memory lymphocytes [4]. Consequently, the interplay between HIV-1 infection in macrophages and macrophage-like cells is markedly less defined. Additionally, the relationship between *in vitro* observations and *in vivo* dynamics is not fully elucidated. Much evidence exists to support the existence of HIV-1 replication in macrophage/macrophage-like cells *in vivo *[5–11], including a recent report from Deleage et al., and confirmed the presence of HIV-1 in macrophages within seminal vesicles of patients on effective highly active antiretroviral therapy (HAART) [12]. Correspondingly, a variety of studies have presented evidence that monocytes harbor productive viral replication in patients receiving HAART [13, 14], with other reports demonstrating that CD16^+^ monocytes, a subset of monocytes, are a source of HIV-1 permissive cells that preferentially harbor HIV-1 *in vivo* [15]. Complementary to these findings, a recent report by Spivak and colleagues demonstrated that circulating monocytes do not harbor latent HIV-1 in elite controllers [16], and an additional finding from Ortiz et al. demonstrated the presence of SIV originating from nonlymphocytic compartments in CD3-depleted rhesus macaques [17]. Despite these findings, they did report the presence of HIV-1 in CD4^+^ T cells in some patients receiving HAART. Together, these studies correlate *in vitro* hypotheses with *in vivo* evidence implicating macrophages as key modulators in viral persistence and warrant further studies designed to fully elucidate this relationship. 

As macrophages are found in diverse tissues that are often independent microenvironments, systemically, and function largely in innate immunity and subsequent antigen presentation to CD4^+^ T lymphocytes in adaptive immunity, their cell cycle and metabolism are clearly distinct from that observed in the activated, proliferating CD4^+^ T lymphocyte. 

Significantly lower levels of dNTP in macrophages than observed in T lymphocytes (Table 1) [18, 19] present a macrophage cellular environment that harbors extremely limited dNTPs, but still high rNTPs (Table 2). This extreme disparity between dNTP and rNTP pools in macrophages can promote preferential incorporation of rNTP into the growing viral DNA strand [19]. Furthermore, understanding which nucleotides present with the highest concentrations in macrophages, which is often distinct and independent from that observed in lymphocytes, serves to facilitate a more robust mechanistic understanding of nucleotide incorporation to be drawn upon in nucleoside analogue drug design. It is now known that the meager macrophage nucleotide dNTP pool is shaped by the macrophages/monocyte restriction factor, SAMHD1, whose triphosphohydrolase activity reduces intracellular dNTP to concentrations that are suboptimal for HIV-1 RT-mediated viral DNA synthesis [20, 21]. 

Although levels of dNTP and rNTP and ratios have been elucidated in macrophages, the impact of preferential rNMP incorporation in macrophages has only recently been explored. Recent reports demonstrate that a concomitant lack of monoribonucleotide repair machinery in these cells, and pausing during DNA synthesis (which is a known correlate of mutagenesis), may point to viral mutagenesis [22]. 

The cellular milieu of macrophages presents with multiple facets that are specific to these cells, all of which comprise a unique microenvironment wherein concentrations of dNTPs and rNTPs orchestrate a complex relationship between HIV-1 and individual or distinct populations of macrophages. Much of these data have been compiled with the use of *in vitro* monocyte-derived macrophages, which represent an excellent tool to model potential *in vivo* dynamics of macrophages found in various microenvironments, although differences between an *in vitro* system and that observed in humans could exist. Nonetheless, compiling a detailed understanding of this interplay can provide a foundation from which to exploit macrophage-specific factors to achieve targeted elimination of HIV-1 from these cells. 

## 2. dNTP Levels in Macrophages: Affecting HIV-1 Reverse Transcription

Lentiviruses possess the unique ability to replicate in nondividing and terminally differentiated cells, unlike many other viruses including oncoretroviruses [23]. The manner in which this is accomplished, and the complex and multifaceted mechanisms that are employed to achieve productive viral replication in nondividing cells, is unique from that observed in activated dividing cells, such as T lymphocytes. 

It is well established that activated, proliferating cells possess significantly higher levels of endogenous dNTPs, which are required for ongoing cellular chromosomal replication in an activated and dividing cell. It follows that dNTP concentrations in T cells are 6–133-fold higher in lymphocytes compared to macrophages, independent of the T cell or macrophage activation state (Table 1) [18, 19, 21, 24], as macrophages are terminally differentiated nondividing cells. 

HIV-1 replication requires a basal level of dNTP to be present to facilitate efficient production of proviral DNA, and without sufficient dNTP levels, productive viral replication occurs suboptimally [24]. Despite significantly lower levels of dNTP in macrophages versus lymphocytes (Table 1), HIV-1 replication is able to proceed due to the uniquely high affinity of HIV-1 RT for its substrate, which facilitates its function.

Viral replication kinetics are delayed in macrophages versus CD4^+^ T cells, and is thought to be a direct function, at least in part, of lower levels of dNTPs available in these cells. Addition of deoxynucleosides (dNs) to the extracellular culture medium, which elevates cellular dNTP concentrations, significantly increases the rate of viral reverse transcription in HIV-infected primary human macrophages, indicating that low levels of dNTPs are a rate-limiting step in the production of HIV-1 [24, 26, 27]. Additionally, the Michaelis constant [28] for dNTPs is low, allowing for efficient binding despite lower overall levels of dNTPs in macrophages (Table 1). This low *K*_m_ is thought to be a result of enzymatic adaptation of viral RT to infect macrophages, allowing for efficient catalysis of viral DNA synthesis despite the low dNTP levels present in these cells [24, 26].

## 3. Levels of dNTPs and Impact on Relative Rate of Incorporation in Macrophages

dNTPs are significantly lower in macrophages compared to T lymphocytes. Despite the low levels of dNTPs in macrophages, the growing viral DNA strand maintains the ability to incorporate selective dNTPs in a concentration responsive manner. For example, noncanonical dUTP concentrations in macrophages is approximately 60-fold higher than that of TTP in macrophages, but is similar to TTP in lymphocytes. Biochemical simulation studies revealed that dUTP is efficiently incorporated into the growing viral DNA strand in the macrophage but not T-cell dNTP environment, suggesting that levels of dNTPs may in part effect which dNTP is incorporated [29]. 

Although increased levels of one dNTP relative to another (e.g., higher levels of dUTP versus lower levels of another dTTP) could confer preferential incorporation, increased levels could also mask differences in *K*_m_, which could also represent a contributing factor in incorporation of dNTP into the growing viral DNA strand. Analysis of pre-steady state and steady state kinetics of dUTP incorporation demonstrated that there is minimal selectivity of HIV-1 RT for TTP compared to dUTP, eliminating the potential for selectivity for substrates as a key factor in frequency of dUTP or TTP incorporation into the HIV-1 proviral genome. It was also demonstrated that 2,3-dideoxyuridine, a specific inhibitor of dUTP incorporation, confers anti-HIV activity in macrophages, but not T lymphocytes, further underscoring the hypothesis that higher levels of dUTP result in preferential incorporation of dUTP as opposed to other dNTPs in HIV-infected macrophages but not in lymphocytes [29]. Overall, dNTP levels and the lack of dUTP/dTTP discrimination are what determine incorporation frequency of dUTP into HIV-1 proviral DNA. The observed antiviral potency of 2′,3′-dideoxyuridine in macrophages provides a proof of principle concept wherein nucleoside analogues could be designed to target inhibition of specific nucleotides in a cell-specific manner, especially with respect to targeting of macrophage-derived viral sanctuaries.

## 4. Cellular Factors and Regulation of dNTP Levels: Host Defense to HIV-1 Infection in Macrophages 

Although lower levels of dNTPs in macrophages and macrophage-like cells, including dendritic cells, are thought to be a key modulator of inefficient viral replication in these cells, the distinct variation between cellular milieus in macrophages in contrast tolymphocytes has led to speculation that a cellular factor unique to macrophages could also contribute to differences in replication kinetics in macrophages versus lymphocytes. 

**Figure 1:**
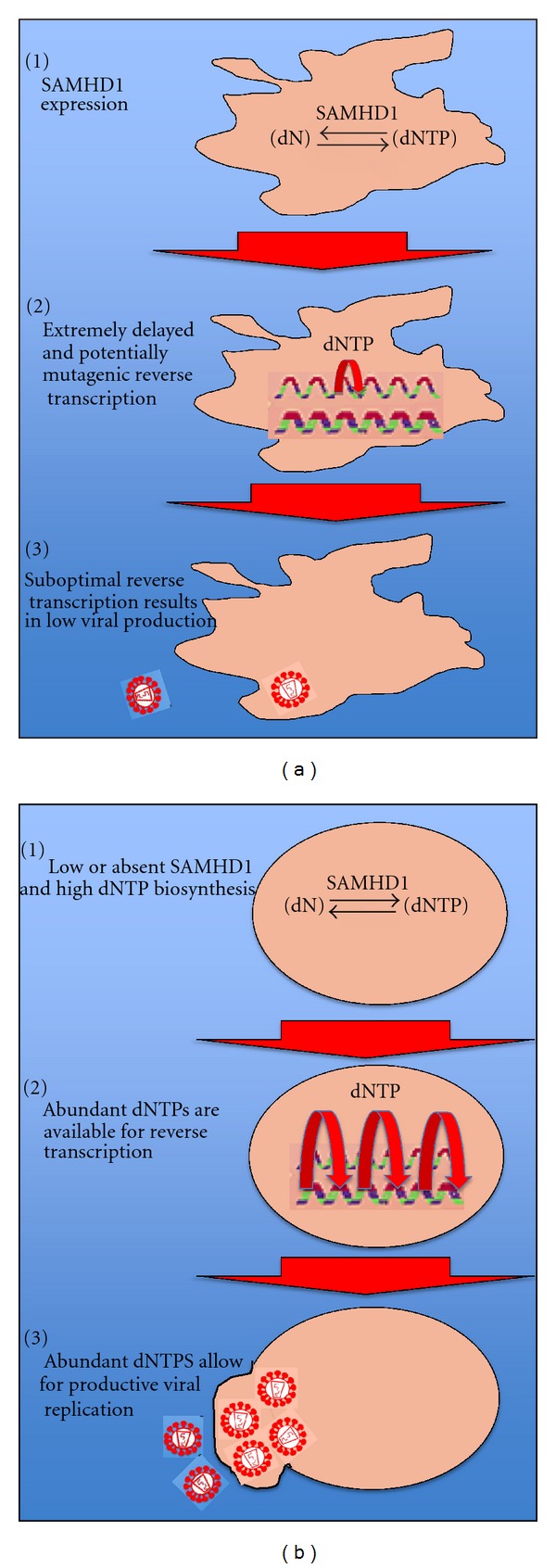
SAMHD1 (SAM domain and HD domain-containing protein 1) and its regulatory mechanism of dNTPs as a host restriction mechanism to prevent HIV-1 infection in macrophages/macrophage-like cells. SAMHD1 cleaves dNTPs into a nucleoside and a triphosphate, rendering levels of intact dNTPs suboptimal to facilitate HIV-1 RT mediated DNA synthesis (a), but low SAMHD1 expression in lymphocytes prevents SAMHD1-mediated restriction in dividing cells such as activated CD4 T cells (b) [20].

Recent reports have identified the sterile alpha motif (SAM) domain and HD domain-containing protein 1 (SAMHD1) protein, which is encoded by the SAMHD1 gene, as a cellular factor that regulates cell-specific restriction of HIV-1 replication in cells of the myeloid lineage [20, 30, 31]. Recent work has shown that reducing the level of dNTPs results in inefficient HIV-1 replication in monocytes/macrophages, and SAMHD1 was identified circuitously by further analysis of the Vpx-mediated enhancement of SIV infection in its natural hosts, and the observed enhanced SIV infection rates in myeloid cells [20]. These data led to the hypothesis that a cellular factor unique to monocyte/macrophage cells could exist and may be modulated by Vpx resulting in the observed enhancement of SIV infection in macrophages but not lymphocytes [31]. SAMHD1 functions as a host restriction factor, to efficiently block viral replication in macrophages and dendritic cells by hydrolyzing cellular dNTPs to a nucleoside and a triphosphate further limiting the pool of available dNTPs for incorporation into the proviral genome (Figure 1). When comparing HIV-1 replication efficiency, the rank order is lymphocytes > macrophages > dendritic cells. It follows that SAMHD1 levels are inversely proportional, wherein SAMHD1 levels are dendritic cells > macrophages > lymphocytes, demonstrating a correlation between SAMHD1 levels and inefficient viral replication [31]. In SIV_sm_ and HIV-2 infections of their natural hosts, the cellular restriction of SAMHD1 is counteracted, as Vpx prevents the SAMHD1-mediated hydrolysis of dNTPs in macrophages, allowing for more efficient viral replication in these cells [30]. The identification of SAMHD1 as a myeloid-specific restriction factor that could provide host-derived protection against infection provides an exciting foundation from which to launch further studies not only about the role of SAMHD1 in modulation of infection in macrophages, but about how controlled interference of imbalanced and scarce dNTPs in HIV-1 target cells could provide a protective measure against infection. 

## 5. dNTP/rNTP Levels in Macrophages: Novel Mechanism for Viral Replication in Macrophages

It is not an unexpected finding that levels of dNTPs are lower in macrophages, which are nondividing terminally differentiated cells, however recent reports elucidated a previously unknown milieu in macrophages relative to ratios and levels of dNTP : rNTP versus that observed in lymphocytes [18, 19]. With respect to delineation of function between dNTPs and rNTPs, dNTPs are primarily a component of chromosomal replication and DNA damage repair, whereas rNTPs perform other functions including substrates for RNA polymerases, metabolic energy carriers, and substrates for a variety of enzymes involved in signal transduction cascades [21, 32]. Therefore, it follows that the levels of rNTP may not be lower in macrophages strictly as a function of the fact that they are nondividing cells, or because dNTP levels are lower in this cell type. 

Recent reports confirmed that although dNTPs are 6–133-fold lower in macrophages versus lymphocytes, independent of activation state, levels of rNTP are only 4–7-fold lower in macrophages (Tables 1 and 2) [18, 19]. These reports were complemented by the finding that rNTP are preferentially incorporated into proviral DNA in the macrophage but not the lymphocyte dNTP : rNTP simulated microenvironment in a biochemical simulation assay [19], a finding that is distinct from the previously accepted dogma that dNTP are incorporated into the growing viral DNA strand exclusively. This report demonstrates that rNTPs are incorporated into the proviral DNA strand in macrophages and also predicts that ribonucleoside chain terminators could be specific inhibitors of HIV-1 replication in macrophages, wherein their mechanism of action would be dictated by the unique landscape of dNTP : rNTP found in macrophages. These data do not exclude the fact that dNTPs are incorporated into HIV-1 proviral DNA, as dNTP-based inhibitors demonstrate anti-HIV activity in macrophages, although potency is diminished for most nucleoside reverse transcriptase inhibitors (NRTI), versus lymphocytes [2, 33, 34]. The fact that rNTP are preferentially incorporated into the growing viral DNA strand in the biochemical simulation of the macrophage cellular environment could in part be responsible for the fact that deoxy-based NRTI are not as potent in macrophages compared to lymphocytes, where dNTPs are preferentially incorporated, especially with respect to chronic infection [2, 33, 34]. 

Last, these data imply that ribonucleoside inhibitors could demonstrate potency against HIV-1 in macrophages, and a recent report confirmed this hypothesis, demonstrating that two ribonucleoside inhibitors, determined to be chain terminators, inhibit HIV-1 RT-mediated DNA synthesis in a dose dependent manner [25]. Together, these data underscore the importance of differences in the macrophage landscape versus lymphocytes, and define for the first time that ribonucleoside inhibitors represent a novel class of antiretroviral therapy that can specifically target HIV-1 replication in macrophages.

## 6. rNMP Incorporation and Implications Relative to Emergence of Mutagenic HIV-1 from Macrophages

Although dNTPs are frequently incorporated into DNA, two phosphate groups are cleaved, with the resulting energy used to create the phosphodiester bond that functions to attach the single remaining phosphate to the growing DNA strand. Therefore, upon discovery that rNTP are preferentially incorporated into the growing viral DNA strand in macrophages, it follows that the incorporated rNTP may undergo the same biphosphate cleavage, potentially resulting in incorporation of rNMP into the growing viral DNA strand in macrophages. 

**Figure 2:**
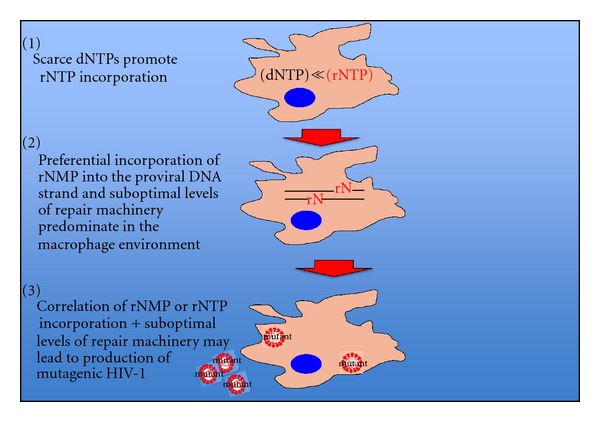
Potential impact of levels/ratios of dNTP : rNTP in macrophages upon emergence of mutagenic HIV-1. Similar ratios of dNTP : rNTP (point 1) confer preferential incorporation of rNTP and rNMP into the growing viral DNA strand (point 2). Together, with suboptimal levels of repair machinery found in macrophages, these incorporations are a known correlate for production of mutagenic HIV-1 (point 3).

Recent reports demonstrate that rNMP is incorporated into HIV-1 proviral DNA, as determined by the presence of 2 LTR circles with quantitative real-time PCR, at a rate of 1/146 nucleotides in macrophages. Additionally, macrophages possess significantly diminished capacity for repairing monoribonucleotides versus that observed in activated lymphocytes, and rNTP incorporation in the template strand preceding the 3′ terminus causes pausing during DNA synthesis, which is a known correlate of mutagenesis. Taken together, the presence of rNMP in the HIV-1 proviral genome, suboptimal levels of repair machinery to remove rNMP in macrophages versus activated lymphocytes, and the established correlation between site-specific incorporation and pausing in DNA synthesis, provide an environment in macrophages that could be a source for increased production of mutagenic HIV-1 [35, 36]. Additionally, it has been previously determined that intracellular levels of the active, triphosphorylated form of nucleoside analogues, NRTI-TP, is significantly lower in macrophages versus lymphocytes, and is often not delivered at adequate levels to inhibit viral replication [37]. Suboptimal levels of drug delivered to macrophages could provide selective pressure for emergence of drug resistant HIV-1, and together with the established environment in macrophages that correlates with increased production of mutagenic HIV-1, point to macrophages as a cell-specific microenvironment that could in theory result in emergence of drug resistant HIV-1 (Figure 2). 

rNMP incorporation occurs at a rate of 1/146 nucleotides in the HIV-1 proviral genome in macrophages, and dNTPs are clearly still incorporated, raising questions about the relative impact of rNMP incorporation in macrophages and its systemic implications *in vivo*. Macrophages are found in every tissue and organ, and due to high CCR5 expression, presence in mucosal sites that often confer primary infection, and rapid localization to the site of infection, all represent significant rationale for *in vivo* relevance of rNMP incorporation into the growing viral DNA strand in macrophages. 

## 7. Relationship between Small dNTP/rNTP, Inflammation, and HIV-1 Disease Progression

dNTPs perform a variety of cellular functions, and levels can be increased as a function of chronic activation of the cell, as is the case in activated versus resting lymphocytes, most notably for TTP and dUTP (Table 1). CD4^+^ T lymphocytes are activated by a variety of stimuli, including paracrine and autocrine cytokine stimulation by proinflammatory cytokines, often as a function of interaction with macrophages/macrophage-like cells in the context of MHCII antigen presentation [38, 39]. In a state of chronic hyperactivation, as is hallmarked by chronic HIV-1 infection that orchestrates increased markers of circulating pro-inflammatory cytokines [40, 41], levels of dNTPs in lymphocytes *in vivo* could, in theory be higher than that of a systemic milieu wherein macrophages are not persistently mediating CD4^+^ T cell activation via antigen presentation and crosstalk within tissue specific microenvironments, including the lymph nodes. As the mechanism of action of NRTI is competition with endogenous nucleotides for incorporation into the growing viral DNA strand, an hypothesized macrophage-mediated increase in dNTPs in CD4^+^ T cells could decrease the potency of NRTI in chronically infected patients. Although this interaction has not yet been proven *in vivo*, better understanding of this relationship, and events governing it, could ultimately elucidate key information that could be used to discover immune-based therapies designed to circumvent hyperactivation of HIV-1 target cells.

## 8. Conclusions

Macrophages are ubiquitous, are infected early in HIV-1 infection, express high levels of CCR5 to garner permissivity to infection, and are sites for establishment and maintenance of latent HIV-1 [4]. These attributes, all of which define macrophages as critical to systemic HIV-1 infection, merit exploration and definition of the dynamics between HIV-1 infection and macrophages, and the corresponding relationship to systemic viremia and disease progression. 

Recent work has begun to establish that various cell-specific attributes of macrophages, including levels and ratios of dNTP, rNTP, and the presence of newly discovered cellular factors unique to macrophages/macrophage-like cells, significantly alter the manner in which HIV-1 replicates in these cells. Recent reports have demonstrated that dUTP is preferentially incorporated relative toother dNTPs in macrophages, and that rNTPs in general are preferentially incorporated into the growing viral DNA strand in macrophages, but not lymphocytes. Additionally, the discovery that rNMPs are incorporated at a rate of 1/146 nucleotides in macrophages, coupled with the established markedly diminished repair capability in macrophages, and the correlation with DNA pausing and production of mutagenic DNA provides a complex landscape wherein HIV-1 replication is altered as a function of the target cell in which replication was facilitated. 

These data afford novel insight into previously unknown mechanisms of HIV-1 replication in macrophages, which are currently being used to design inhibitors targeting incorporation of rNTP, rNMP, or dUTP. As current HAART has not been able to eliminate virus from all tissues and reservoirs, it is unlikely that inhibitors of rNTP, rNMP, or dUTP could solely eliminate virus from macrophage-derived reservoirs. However, together this knowledge about dNTP and rNTP incorporation into proviral DNA, and their impact upon HIV-1 infection in macrophages defines a complex landscape and provide a springboard from which to launch a multipronged approach to eliminate virus from macrophage-derived viral sanctuaries. 

## Figures and Tables

**Table 1:** Concentrations of dCTP, dGTP, dATP, TTP, and dUTP in activated or resting primary human macrophages versus lymphocytes. Concentrations of dNTPs are 6–133-fold lower in macrophages versus lymphocytes, independent of activation state [18, 25]. ±indicates standard deviation. Data represents at least five independent experiments performed with pooled cells from six independent donors.

	dCTP	dGTP	dATP	TTP	dUTP
	*μ*M				
Activated lymphocytes	3.7 ± 2.7	1.52 ± 1.01	9.2 ± 4.5	16.0 ± 5.3	12.0 ± 1.8

Activated macrophages	0.15 ± 0.10	0.05 ± 0.03	0.10 ± 0.07	0.15 ± 0.10	2.0 ± 9.5

Fold difference between activated lymphocytes versus activated macrophages	25	30	92	107	6

Resting lymphocytes	4.5 ± 2.9	0.91 ± 0.35	5.3 ± 2.2	2.9 ± 2.0	21.6 ± 0.5

Resting macrophages	0.07 ± 0.05	0.07 ± 0.05	0.04 ± 0.03	0.05 ± 0.04	2.9 ± 1.3

Fold difference between resting lymphocytes versus resting macrophages	64	13	133	58	8

**Table 2:** Concentrations of CTP, GTP, ATP, UTP in activated or resting primary human macrophages versus lymphocytes. Concentrations of rNTPs are only 4–7-fold lower in macrophages versus lymphocytes independent of activation state [18, 25]. ±indicates standard deviation. Data represents at least five independent experiments performed with pooled cells from six independent donors.

	CTP	GTP	ATP	UTP
	*μ*M			
Activated lymphocytes	182 ± 24	1,745 ± 128	6,719 ± 560	690 ± 100

Activated macrophages	27 ± 8	303 ± 60	1,011 ± 247	141 ± 17

Fold difference between activated lymphocytes versus activated macrophages	7	6	7	5

Resting lymphocytes	111 ± 30	923 ± 234	4,753 ± 896	453 ± 174

Resting macrophages	25 ± 8	323 ± 95	1,124 ± 339	173 ± 47

Fold difference between resting lymphocytes versus resting macrophages	4	3	4	3
